# The coevolution of rostral keratin and tooth distribution in dinosaurs

**DOI:** 10.1098/rspb.2023.1713

**Published:** 2024-01-17

**Authors:** Isaura Aguilar-Pedrayes, Jacob D. Gardner, Chris L. Organ

**Affiliations:** ^1^ Department of Earth Sciences, Montana State University, Bozeman, MT 59715, USA; ^2^ School of Earth Sciences, University of Bristol, Bristol BS8 1QU, UK; ^3^ School of Biological Sciences, University of Reading, Reading RG6 6AH, UK

**Keywords:** coevolution, jaw keratin, toothrow, dinosauria

## Abstract

Teeth evolved early in vertebrate evolution, and their morphology reflects important specializations in diet and ecology among species. The toothless jaws (edentulism) in extant birds likely coevolved with beak keratin, which functionally replaced teeth. However, extinct dinosaurs lost teeth multiple times independently and exhibited great variation in toothrow distribution and rhamphotheca-like keratin structures. Here, we use rostral jawbone surface texture as a proxy for rostral keratin covering and phylogenetic comparative models to test for the influence of rostral keratin on toothrow distribution in Mesozoic dinosaurs. We find that the evolution of rostral keratin covering explains partial toothrow reduction but not jaw toothlessness. Toothrow reduction preceded the evolution of rostral keratin cover in theropods. Non-theropod dinosaurs evolved continuous toothrows despite evolving rostral keratin covers (e.g. some ornithischians and sauropodomorphs). We also show that rostral keratin covers did not significantly increase the evolutionary rate of tooth loss, which further delineates the antagonistic relationship between these structures. Our results suggest that the evolution of rostral keratin had a limited effect on suppressing tooth development. Independent changes in jaw development may have facilitated further tooth loss. Furthermore, the evolution of strong chemical digestion, a gizzard, and a dietary shift to omnivory or herbivory likely alleviated selective pressures for tooth development.

## 1. Introduction

Beaks are edentulous structures covered by a keratinous sheath (rhamphotheca) in the outer (rostral) and part of the inner (oral) surfaces of the jaw bones, which are present in the jaws of extant birds and turtles [1]. Because mouths are operationally responsible for food intake and object manipulation, beaks and teeth have undergone antagonistic coevolution [1]. For example, in birds, beaks would be favoured over teeth to lighten the skeleton as a flight adaptation [1–3]. This hypothesis is supported by genetic and developmental biology studies that suggest irreversible tooth loss as the rhamphotheca expands from a simple egg tooth (caruncle) to a full sheath covering both rostral and oral surfaces [4,5]. However, the ecology and life history of extant birds may not reflect those of extinct relatives that independently evolved rhamphotheca-like rostral keratinous structures—such as non-avian theropods, sauropodomorphs and ornithischians. Many avian traits, such as feathers, genomic contraction and hollow bones, were also thought to be adaptations for flight, but research in the last four decades has revealed other functional contexts [6–13].

Mesozoic dinosaurs were tremendously successful—they occupied a diverse range of ecological niches comparable to modern mammals [14,15]. Niche partitioning among sympatric dinosaurs can be inferred by differences in tooth shape, deposition of dentin, tooth replacement rates and jaw mechanics [16]. However, unlike mammals, several dinosaur groups evolved keratinous beaks; the fleshy snouts of monotremes, such as the modern platypus (*Ornithorhynchus anatinus*), resemble bird ‘bills’ but lack a rhamphotheca and only contain oral keratin pads that replace molars in adulthood [17]. In addition, the fossil record of Mesozoic dinosaurs suggests a complex relationship between teeth and rhamphotheca-like rostral keratin structures. Teeth coexist with rhamphotheca-like keratin structures in the same rostral bones of some primitive sauropodomorphs, sauropods and ornithischians. *Chilesaurus diegosuarezi* is another interesting case where a rostral rhamphotheca-like keratin structure might have coexisted with distal premaxillary teeth (Novas *et al.* [18]). However, this species is currently difficult to assess due to the fragmentary nature of the jaws and uncertain position in the dinosaur phylogeny [19–21].

The fossil record of primitive birds and non-avian theropods shows various trends of tooth distribution among species and between the upper and lower jaws. A keratin cover on the rostral tips of the jaws is also observed [1,22], with possible overlap with teeth in rare cases [23]. For example, tooth loss is inferred in the anterior maxillae and dentaries of the oviraptosaurians *Incisivosaurus* and *Protarchaeopteryx* [24–26]. Whereas posterior toothrow reduction of the maxillae and dentaries is observed in many ornithomimosaurs [26,27] and enantiornithine birds [26,28–30], tooth loss in the premaxillae and anterior dentaries are observed in archaic therizinosaurs [26,31–33] and some ornithuromorphan birds [26,34,35]. Differences in toothrow development between the premaxillae and the maxillae of Mesozoic birds (avialans) suggest that toothrow evolution is modular. This could explain the absence of a general trend toward edentulism in the clade [36]. The Cretaceous-Paleogene (K-Pg) mass extinction may have also acted as an ecological filter that favoured edentulous birds [37]. Irreversible tooth loss is acquired after the jaws are edentulous [36]. The latter has been cited as an example of Dollo's Law [1,36,38], where any complex structure lost through evolution is unlikely to be re-acquired in the same form [39].

Dinosaur fossils offer an excellent opportunity to test the influence of rostral keratin on toothrow development. However, the macroevolutionary relationship between rhamphotheca-like keratin and tooth distribution has not been analysed across Dinosauria. We explore this taxonomic diversity to test hypotheses about the evolution and coevolution of teeth and beaks. Statistical correlation can be consistent with *a priori* hypotheses of adaptive trait associations given the proper evolutionary sample size—the number of independent evolutionary shifts in trait values [40]. We assess the evolutionary relationships between both traits using a phylogenetic generalized linear mixed regression model. We reconstruct ancestral states to discern the evolutionary trends of tooth loss preceding keratin cover evolution in the three major dinosaur lineages (Theropoda, Sauropodomorpha and Ornithischia). We also assess the antagonistic selection hypothesis by testing if the presence of rostral rhamphotheca-like keratin increases the evolutionary rate of tooth loss.

## 2. Material and methods

**Figure 2.**
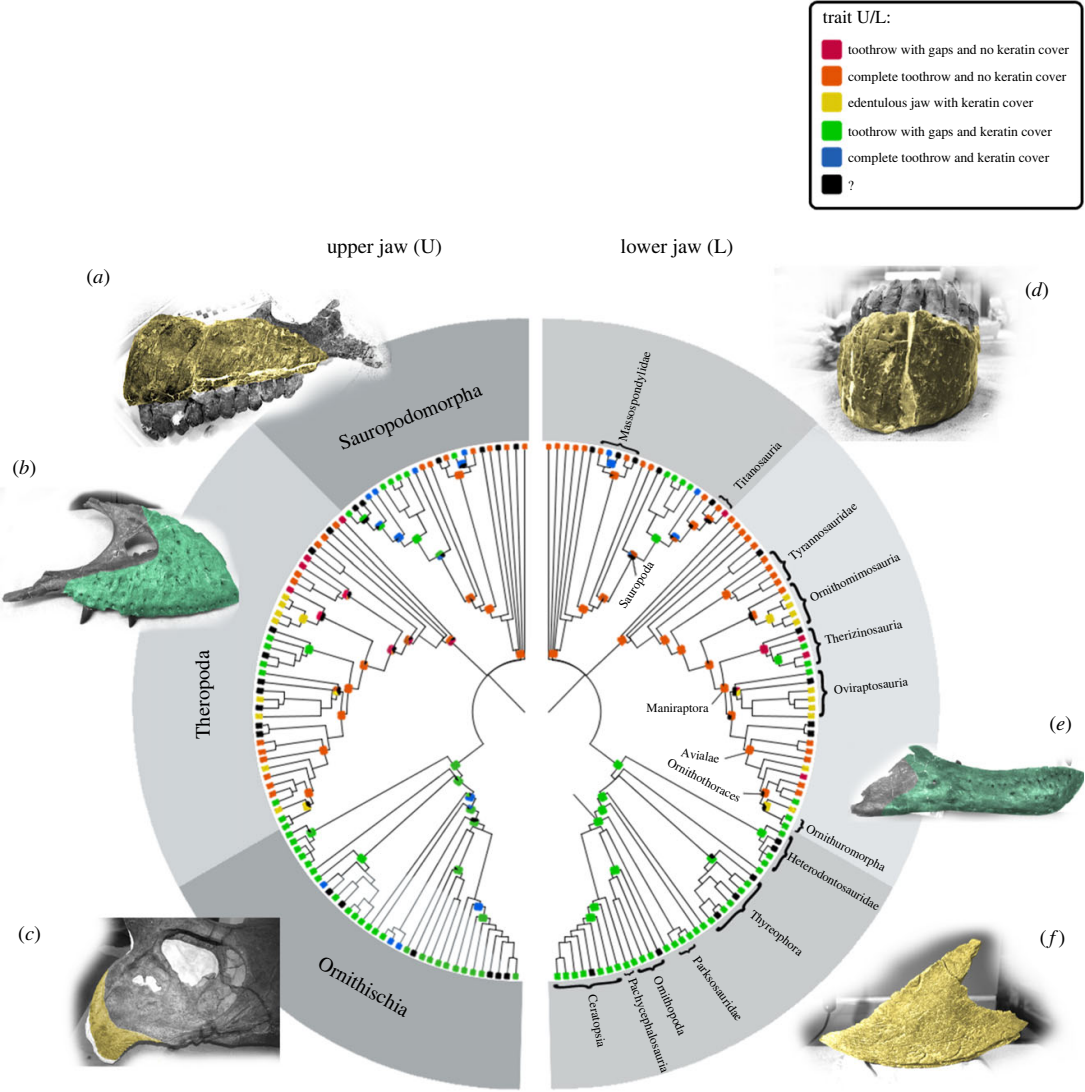
From left to right: ancestral state reconstruction of the upper jaw trait (*U*) and the lower jaw trait (*L*) in Dinosaurs. Missing data or uncertain ancestral states are highlighted in black squares, and trait states are highlighted in squares of five different colours (red, orange, yellow, green and blue) following electronic supplementary material, table S2 (SM1). Phylogeny follows the tree of Button & Zanno [15] using *ape* and *phytools* R packages. The tree excludes most species not included in the project. Photographs of fossil specimens with rostral keratin cover bone proxies highlighted in yellow (*a,c,d,f*) and bone proxies for flat facial scales in green (*b,e*). Camarasaurus BYU 24688 upper jaw (*a*) and lower jaw (*d*). *Tyrannosaurus_rex* MOR-1125 maxilla (*b*) and dentary (*e*). *Triceratops prosus* MOR 1604 upper jaw (*c*) and lower jaw (*f*).

We coded discrete morphological characters relating to the upper and lower jaws for 40 species of theropods, 23 species of sauropodomorphs and 30 species of ornithischian dinosaurs. Specimens used for phylogenetic analyses have rostral surfaces intact for keratin cover inference. The term ‘rostral keratin cover’ is used to refer to jaws with direct or indirect evidence of ‘rostral or outer surface rhamphotheca’, which avoids the ambiguity with a ‘rhamphotheca’ defined as a ‘toothless’ keratinous structure that covers both the rostral and part of the oral surfaces of the jawbones*.* Rostral keratin cover data (KC) was treated as binary (0: absent, 1: present), whereas toothrow condition (TR) was coded as a multistate variable (0: full dentition, 1: partial dentition, 2: edentulous). The grooves located at the rostral tip of jawbones are used as rostral keratin cover proxies (figure 2; electronic supplementary material, figures S1–S3, S5 (SM1)) in fossil jaws following Hieronymus *et al*. [41]. These bone surface proxies have been used to identify missing rhamphotheca in fossils [41]. These bone proxies could be distinguished from the hummocky texture in some specimens (figure 2; electronic supplementary material, figures S3 and S4 (SM1)). The latter has been interpreted as proxies for flat facial scales (figure 2; electronic supplementary material, figures S3 and S4 (SM1)) following Hieronymus *et al*. [41] and Carr *et al*. [42]. Rostral surfaces with only linearly aligned foramina along the jaw margin that are in low densities were also interpreted as surfaces without rostral keratinous cover since this has been associated with lipped jaws in modern squamates [43]. To infer toothrow distribution, we relied on in-place teeth and non-vestigial alveoli [5], which accounts for any teeth missing due to taphonomic filters or collection errors.

We used Button & Zanno's [15] Dinosauria phylogeny for our phylogenetic comparative analyses. Given the uncertainty of the Dinosauria phylogeny in recent studies [44–47], we maintained a base polytomy separating the three main dinosaur lineages, Ornithischia, Sauropodomorpha and Theropoda, at an equal distance.

**Figure 1.**
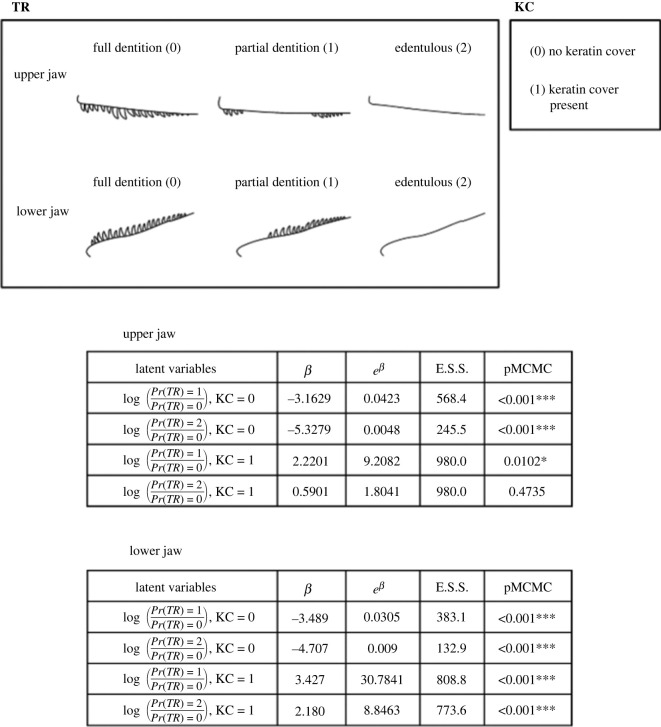
From left to right downwards: trait toothrow (TR), trait rostral keratin cover (KC) and the tables for the fixed effects models for the upper and lower jaws using the imputed dataset. In the latent variable equations, *Pr(TR)* represents the probability of observing a specific toothrow state given the rostral keratin cover state. The tables include the log-odd posterior probability mean (*β*), the probability of one toothrow state (partial dentition or edentulous condition) against the probability for full dentition condition, the mean odds (*e^β^*), the effective sample size (E.S.S.) and the p Markov-chain Monte Carlo (pMCMC) statistic. Each row represents a latent variable (the probability of observing TR = 1 or 2 over TR = 0) associated with each rostral keratin cover state (KC = 0 or 1). * Indicates statistical significance (pMCMC < 0.05) and *** indicates the strongest significance (pMCMC < 0.001).

We used a multinomial regression model [48] to assess if the presence of KC affected the TR of dinosaurs using the MCMCglmm R package [49]. We set the Markov-chain Monte Carlo (MCMC) algorithm procedures samples the posterior distribution of slope parameters for 200 000 iterations (burn-in = 10 000) and 100 sampling frequency. In our model, TR is the response variable and has three states (J). To accommodate over two states in the response, the model creates *J*-1 number of latent variables. Each latent variable (*l_i_*) is a partitioned state of TR (state 1: partial dentition, state 2: edentulous). These partitioned states are compared with a baseline state (state 0: full dentition), the dinosaurian ancestral state. We applied a logit transformation to the response, amounting to a logistic regression on each latent variable. The model will then estimate the log-odds ratio of each TR state being observed over the baseline full dentition state given KC, the explanatory variable, as shown in the equations in figure 1. In the equations, *Pr*(*TR*) represents the probability of observing a specific toothrow state. We assessed statistical significance using the proportion of model parameters that cross 0 (pMCMC).

To account for missing data, we imputed characters using random forest imputation, implemented in the MissForest R package [50]. Prediction of missing biological data was aided by including phylogenetic eigenvectors and imputation quality was measured with the proportion of falsely classified entries (PFCs) following Fournier *et al*. [51] and Penone *et al*. [52]. PFC is a type of out-of-bag error estimation for categorical data.

**Figure 3.**
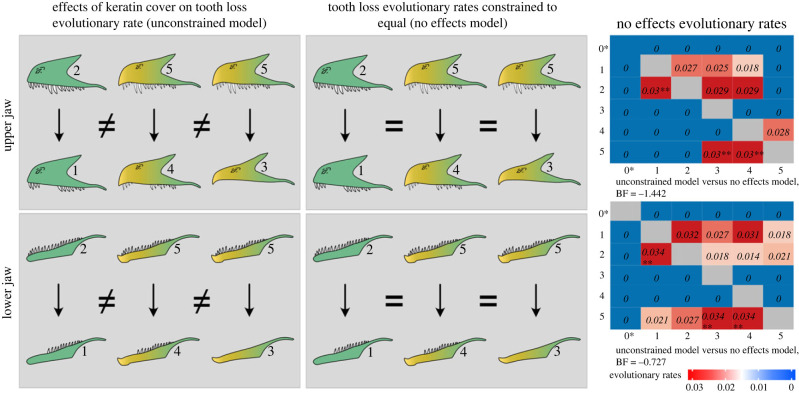
From upper row to lower row: evolutionary transitions for the upper jaw and the lower jaw. From left to right columns: unconstrained RJ-MCMC model on tooth evolutionary rate while accounting for rostral keratin cover presence and absence, hypothetical no effects models on tooth loss evolutionary rate (rates are equal regardless of rostral keratin cover state) and the evolutionary rates results of the no effects model. Trait state classification based on electronic supplementary material, figure S8 of SM1. Colours in jaw figures: green for the absence of rostral keratin cover, and yellow with green to represent the presence of rostral keratin cover without assuming the exact caudal extension of the rostral keratin cover. The location of gaps in toothrow differs between species of dinosaurs. 0* There were no specimens in the original dataset that had edentulous jaws without rostral keratin cover bone proxies. Heatmap shows the different evolutionary rates for changing from one trait state (row) to another trait state (column). ** Represent the evolutionary transitions fixed at equal rates. Evolutionary transitions that are equal (=), evolutionary rates that are different (≠). Bayes factor (BF) values does not support the unconstrained RJ-MCMC models compared with the no effects model (BF > 2 indicates positive evidence favouring the alternative model).

We reconstructed ancestral states and estimated evolutionary rates using a reversible-jump Markov-chain Monte Carlo (RJ-MCMC) algorithm in the program BayesTraits V3.2.6 (http://www.evolution.rdg.ac.uk). This analysis was conducted for all Dinosauria and the three dinosaur lineages separately. All combinations of states for keratin cover and toothrow condition were recoded as a single multistate trait for the upper jaw (*U*) and lower jaw (*L*) separately (electronic supplementary material, figure S8 and table S2 (SM1)). We used Bayes factor (BF) tests for model selection based on Raftery *et al*. [53] to test if keratin cover affects rates of toothrow evolution. Here, we compared an RJ-MCMC unrestricted evolutionary rates model against the null hypothesis model of equal evolutionary rates for tooth loss regardless of rostral keratin cover presence (figure 3). A BF > 2.0 indicates positive support for the model with the highest log marginal likelihood. Bayesian *t*-tests were performed to determine whether there were significant differences between the evolutionary rates of the upper jaw and lower jaw. Further details on our methods can be found in electronic supplementary material, SM1.

## 3. Results

The multinomial regression tests with imputed data (figure 1) strongly support the hypothesis of toothrow reduction if rostral keratin cover is present. However, rostral keratin cover presence is more likely associated with partial toothrow reduction than complete jaw tooth loss. When rostral keratin cover is present (KC = 1), the mean odds of having toothrow gaps is 9.21 times more likely than having full dentition in the upper jaw (e*^β^* = 9.21; pMCMC = 0.01). This pattern is even stronger in the lower jaw (mean odds, e*^β^* = 30.78; pMCMC < 0.001). If there is rostral keratin cover, the mean odds of having an edentulous upper jaw is 1.80 times more likely than having full dentition in the upper jaw. Although, this estimate is not statistically significant (pMCMC = 0.47). This result differs from the lower jaw, where an edentulous condition is 8.85 times more likely to be associated with a rostral keratin cover (e*^β^* = 8.85; pMCMC < 0.001). The distinction between the upper and lower jaws may reflect uneven selection pressures on different parts of the jaws. The analyses without imputed data yielded similar results (electronic supplementary material, table S2).

The RJ-MCMC ancestral state reconstructions (figure 2; electronic supplementary material, tables S3–S8) showed that only the theropod lineage had prior toothrow reduction before evolving rostral keratin cover (e.g. in the lower jaws of therizinosaurs). Jaws with rostral keratin cover and partial toothrows are widespread in the ornithischian lineage. Still, our discrete character coding masks the spatial heterogeneity of toothrow gaps. Our ancestral state reconstructions also reveal examples of toothrow gap reversal in both ornithischian and sauropodomorphan lineages. In other words, continuous toothrows can evolve from toothrows with gaps.

Our analysis did not detect (BF < 2) an effect of rostral keratin cover on the rate of toothrow evolution among all dinosaurs (figure 3; electronic supplementary material, table S10). In addition, under the no effects models, we found no evidence for a difference in the overall evolutionary rates of the upper and lower jaws (pMCMC > 0.05). The difference between the evolutionary rates of the lower and upper jaws is less than 1 × (0.005 times), which suggests that the rates were similar (electronic supplementary material, table S20). However, there are detectable differences in the individual rate parameters between the upper and lower jaws (figure 3; electronic supplementary material, tables S11–S12). The rate of acquiring a continuous toothrow increases for the upper jaw if a rostral keratin cover is present; however, we do not observe this effect in the lower jaw. By contrast, the rate of acquiring rostral keratin cover with no change to the full dentition condition is higher in the lower jaw than the upper jaw (transition rate from state 2 to 5 = 0.021 in the lower jaw and 0 in the upper jaw; figure 3). Similar results were seen in analyses on Theropoda, Ornithischia and Sauropodomorpha (electronic supplementary material, tables S13–S18, S20).

## 4. Discussion

Previous studies have found a negative relationship between teeth and rhamphotheca development in birds and closely related non-avian theropods [4,5]. However, this conclusion may be influenced by the physiological, embryological, and ecological characteristics of extant birds. To address these potential biases, we include non-avian dinosaurs (Theropoda, Sauropodomorpha, and Ornithischia) in our analysis and utilize phylogenetic comparative methods. Our goal is to investigate the impact of rostral jaw keratin cover on the evolution of complete and partial tooth loss. Our findings support the hypothesis that the reduction of toothrows in dinosaurs, including Mesozoic birds, was driven by the evolution of rostral jaw rhamphotheca-like keratin. Nonetheless, the analyses conducted in this study do not provide an explanation for complete tooth loss, especially in the upper jaw.

### (a) The complex evolutionary relationship of tooth loss and rhamphotheca-like keratin structures

The coevolution of tooth loss and the presence of keratin similar to the rhamphotheca in dinosaurs is a more intricate process than a simple antagonistic relationship. Our multinomial logistic model confirms that rostral keratin cover has an impact on partial tooth loss, but not on complete tooth loss. We would anticipate that the chances of complete tooth loss would rise with rostral keratin cover, but we do not observe this effect in the upper jaws. In addition, our RJ-MCMC models also support the reversal of toothrow gaps when there is a rostral keratin cover present in the upper part of the jaw. This applies to theropods, sauropodomorphs, ornithischians, and dinosaurs as a whole.

The independent evolution of rostral keratin covers and tooth patterns in dinosaurs might indicate alternative molecular interactions during development. Retaining distal teeth on the jaws might have affected variation in the toothrow distributions of sauropodomorphs and ornithischians. These teeth could have halted distal to proximal oral expansion of the rostral jaw keratin cover or, in addition to the rostral keratin cover, made it impossible for an independent oral keratin structure to evolve and replace teeth. In contrast, true beaks, which have a rhamphotheca that covers both the rostral surface and part of the oral surface of the edentulous jawbones, evolved later in some dinosaur groups (e.g. Ornithuromorpha, Oviraptosauria, Ornithomimosauria, Ceratopsia, Ornithopoda). Experiments with modern bird and turtle embryos show that the Shh (Sonic the Hedgehog protein) gene, alongside other genes, is needed to form the tooth-forming region prior to tooth development [54–56]. However, most of the genes needed for enamel growth are absent (enamelin [ENAM] and amelogenin [AMBN]) in modern birds, whereas the gene for dentine formation (dentin sialophosphoprotein [DSPP]) is inactive [54,55]. Studies of beak development in bird embryos suggest that the cellular and molecular mechanisms involved in tooth and rhamphotheca formation have direct antagonistic interactions [5,57]. In particular, BMP4 (bone morphogenetic protein 4) regulates the shifts in temporal gene expression of the neural crest section that determines the shape and expansion of the keratinous caruncle and rhamphotheca. BMP4 overexpression might have caused early odontogenic arrest in beaked theropods, which in turn may have caused the deletion and functional loss of genes needed for odontogenesis through genetic mutation. In contrast, early odontogenic development arrest in turtle embryos is caused by termination of Msx2 (homeobox protein 2) expression in the dental mesenchyme [56].

### (b) Evolutionary differences between dinosaur lineages

Teeth have multiple functions, including holding, piercing, slicing, and grinding. Beaks have similar functions [1], but they usually lack the ability or have limited ability to mechanically break down food [58]. Our research reveals that the evolutionary relationship between tooth loss and rhamphotheca-like keratin varies among dinosaur clades. If there was a simple antagonistic relationship, we would expect the evolutionary trends to be consistent across all dinosaurs. Additionally, if rhamphotheca-like keratin provided an adaptive advantage over teeth, we would anticipate higher rates of toothrow reduction in dinosaurs with a rostral keratin cover. The rostral keratin cover would rapidly expand along the jawline, while the functional redundancy of teeth would lead to tooth loss. However, this does not appear to be the case. Dinosaur lineages without rostral keratin covers were just as likely to lose teeth as those with rostral keratin covers.

According to Brocklehurst & Field [36], there was no overarching trend towards edentulism in avialans, which includes modern birds. Still, partial tooth reduction occurred before the evolution of the rhamphotheca. Our analyses suggest that many theropod groups independently reduced toothrows before evolving beaks. In contrast, ornithischians and sauropodomorphs reduced toothrows but not before evolving rostral keratin covers. Some ornithischian groups (e.g. Agilisaurus louderbacki, Jeholosaurus shangyuanensis, Yinlong downsi) and sauropodomorph groups (e.g. Adeopapposaurus mognai, Abydosaurus mcintoshi, Camarasaurus lentus) also gained complete toothrows from partial dentition despite having rostral keratin covers.

A strong shared function constraint between the upper and lower jaws explains the similarity in the overall rates of evolution of both jaws in dinosaurs, particularly in terms of toothrow development and the presence of keratin cover. Feeding habits impose similar functional constraints on both the upper and lower jaw. However, there are some consistent differences in the rates of evolution between the two jaws. The evolution of toothrow variation after the appearance of rostral keratin cover was more flexible in the upper jaw compared to the lower jaw. This is supported by our RJ-MCMC models, which show high rates of toothrow reduction and toothrow gap reversal in the upper jaw with rostral keratin cover, while there is no rate change for these same evolutionary transitions in the lower jaw.

Button & Zanno [15] detected two evolutionary modes of herbivory in dinosaurs, which included gracile cranial, low bite forces and extended gut processing in theropods and sauropodomorphs, with ornithischians developing several cranial characters associated with extensive oral processing. They concluded omnivory was likely ancestral in dinosaurs. Our dinosaur ancestral reconstruction suggest that tooth row states followed this pattern. In ornithischians, oral processing specialization constrained the jawline to different jaw regions using beaks and teeth simultaneously. Beaks can clip food, while teeth can grind it [59,59]. The mammal heterodont dentition is analogous to this jaw configuration. A greater reliance on gut processing in the common ancestor of Theropoda and Sauropodomorpha might explain our results, which show a greater variation in the evolutionary relationship between rostral keratin covers and toothrows than in ornithischians. Carnivory demands jaw specialization for fast prey capture at the expense of efficiency in food processing in dinosaurs and squamates [60,61]. Consequently, theropod gizzards, like in modern birds, could have taken the exclusive role of grinding food while teeth immobilized prey [62]. Under this hypothesis, a shift towards herbivory in theropods would have facilitated complete tooth loss. Biomechanical tests on the skull of Erlikosaurus andrewsi, a herbivorous theropod, show that replacing distal teeth with a keratinous rhamphotheca helps reduce stress and strain, making the rostral part of the skull less susceptible to bending and displacement [33].

In addition, an early shift towards herbivory and increased body size in sauropodomorphs might have further relaxed the functional selective pressure that controlled the relationship between rostral keratin covers and toothrows. For example, the rostral keratin cover could have functioned as ornamentation for sexual or interspecific signalling, and (or) as a protective barrier for bulk feeding on abrasive plant matter. Diplodocid sauropods, such as Camarasaurus, Diplodocus, Europasaurus, and Nigersaurus, have no or reduced bony septa that separate teeth into discrete tooth alveoli and exhibit tooth root reabsorption, like in Camarasaurus [63–65]. Sauropod keratin cover and thick gingival tissue might have held these teeth in place while allowing for higher tooth replacement rates [64,65].

This way, sauropods could bulk feed on tough, abrasive plant matter that wore down teeth. Small rostral keratinous plates at the tips of the upper and lower jaws might have evolved independently from sauropods in the ancestor of the non-sauropod sauropodomorphs *Leyesaurus* and *Adeopapposaurus* for clipping foliage.

Our evolutionary rate models indicate that there is no loss of keratin cover in the front part of the upper jaw. However, in the lower jaw, the loss and gain of keratin cover in the same area can occur at similar rates when there is no change in the continuous toothrow condition or when there is a transition from a toothrow with gaps to a continuous toothrow. Nevertheless, the loss of keratin cover in the lower jaw is highly unlikely due to the relatively low rates mentioned, and it is not evident in our ancestral reconstruction of dinosaur jaws.

### (c) Considerations and future work

Future research could expand on our findings by examining the distribution of ornithischian toothrows in the upper jaw, which is more varied compared to the lower jaw. Unfortunately, we did not consider this distinction in our character coding. It is worth noting that only sauropods among all dinosaur groups possess a significant region on their tooth-bearing jawbones that could be indicative of rostral keratin cover proxies. However, the areas of the jaws that experience tooth loss rarely exhibit these keratin cover proxies. Unfortunately, our character coding does not account for this as well. To address these limitations, principal component analyses (PCA) could be employed to simplify the multidimensional data and potentially offer a solution.

Phylogenetic parameters, such as ancestral states and evolutionary rates, are difficult to estimate accurately when working with small evolutionary sample sizes [40]. Small sample sizes result in low statistical power and increase the likelihood of biased parameter estimations [67,68]. For instance, the absence of basal taxa may explain why intermediate steps of tooth reduction leading to the evolution of keratin cover in ornithischians and most sauropodomorphs have not been observed. Conducting future research with better sampling could help elucidate the differences observed among the three main dinosaur lineages. Additionally, investigating the evolutionary relationship between teeth and rhamphotheca-like keratin structures in comparison to other archosaurs, such as pterosaurs and crocodylomorphs [1], known to have independently evolved beaks, would be valuable. Another hypothesis to explain the evolution of 'beak' and teeth in sauropodomorphs is that the rostral grooves with pits do not serve as proxies for a rhamphotheca-like structure. As explained by Martínez [69], similar osteological features have been observed in the jaws of extant mammals, such as hippopotamuses and manatees, which have highly mobile lips. It is believed that non-avian dinosaurs did not possess facial muscles associated with mobile or prehensile lips. Wiersma & Sanders [66] suggested that the jaws of sauropods, like Camarasaurus, could have been covered by thick gingival connective tissue and scales or a rhamphotheca-like structure on top. In order to differentiate between these rostral tissues, researchers will need to examine other external and internal landmarks in extant animals.

## 5. Conclusion

We find that rostral keratin cover influences partial tooth reduction but does not explain complete tooth loss in dinosaurs. Importantly, the evolution of beak-like rostral keratin covers did not lead to increasing rates of tooth loss, suggesting that rhamphotheca-like keratin structures did not antagonistically select against the presence of teeth. Future research with better taxonomic sampling of basal dinosaurs (and related non-dinosaurian groups) could shed light on the differences detected among the three main dinosaur lineages (Theropoda, Sauropodomorpha and Ornithischia). The spatial influence of rhamphotheca-like structures on toothrow evolution could be studied using morphometric and biomechanical methods, such as phylogenetic principal component analysis. Other bone proxies should be explored to distinguish rhamphotheca-like keratin structures from other rostral tissues.

## Data Availability

The dataset (SM3.xlsx) supporting this article have been uploaded into DRYAD. https://datadryad.org/stash/share/ic2knyDYGUy1H4UgKcydiHJhNO2OLyypZEA_ErBdorU. Dataset citation with DOI: Aguilar Pedrayes, Isaura; Gardner, Jacob D.; Organ, Chris L. (Forthcoming 2023). The coevolution of rostral keratin cover and toothrow distribution in Mesozoic Dinosaurs (Dataset). Dryad. https://doi.org/10.5061/dryad.7pvmcvf14 [70]. Electronic supplementary material (SM1 and SM2) is submitted along with the manuscript. The datasets supporting this article have been uploaded into Zenodo (https://doi.org/10.5061/dryad.7pvmcvf14) [71], and extensions of methods and results as the electronic supplementary material (SM1 and SM2) is provided along this paper. Supplementary material is available online [72].
